# Molecular mechanisms underlying nutrient detection by incretin-secreting cells

**DOI:** 10.1016/j.idairyj.2009.11.014

**Published:** 2010-04

**Authors:** Frank Reimann

**Affiliations:** Cambridge Institute for Medical Research, Wellcome Trust/MRC Building, Addenbrooke's Hospital, Hills Road, Cambridge CB2 0XY, UK

## Abstract

The hormones glucose-dependent insulinotropic polypeptide (GIP) and glucagon-like peptide-1 (GLP-1) are secreted postprandially from intestinal K- and L-cells, respectively. As incretins, these hormones stimulate insulin secretion from the pancreatic β-cell, and have independently been implicated in the control of food intake and lipid metabolism. Whilst the enteroendocrine cells producing GIP and GLP-1 are therefore attractive targets for the treatment of diabetes and obesity, our understanding of their physiology is fairly limited. The mechanisms employed to sense the arrival of carbohydrate, fat and protein in the gut lumen have been investigated using organ perfusion techniques, primary epithelial cultures and cell line models. The recent development of mice with fluorescently labeled GIP or GLP-1-expressing cells is now enabling the use of single cell techniques to investigate stimulus-secretion coupling mechanisms. This review will focus on the current knowledge of the molecular machinery underlying nutrient sensing within K- and L-cells.

## 1 Introduction

The term “incretin effect” describes the observation that orally ingested glucose stimulates insulin secretion more potently than intravenously administered glucose, even when plasma glucose excursions are matched (Creutzfeldt, 1979; Elrick, Stimmler, Hlad, & Araim, 1964). The effect originates from two hormones, glucose-dependent insulinotropic polypeptide (GIP) and glucagon-like peptide-1 (GLP-1), which are secreted from enteroendocrine cells embedded in the gut epithelium. These so-called “incretins” are believed to act directly on the pancreatic β-cell, which expresses specific G-protein coupled receptors (GPRs) for both these hormones (Baggio & Drucker, 2007). Receptor stimulation increases cytosolic concentrations of cyclic adenosine monophosphate (cAMP), which in turn boosts insulin secretion, albeit only under permissive conditions when secretion is simultaneously initiated by elevated blood glucose levels (Gromada, Holst, & Rorsman, 1998). The resulting relatively small risk of inducing hypoglycaemia, and the observation that some of the recently introduced incretin based medications for type-2 diabetes are associated with weight loss, are major advantages over older insulinotropic medications, such as the sulphonylureas. Current drugs that exploit the GLP-1 axis are based either on GLP-1 mimetics or on inhibition of dipeptidylpeptidase IV (DPP-IV), which normally rapidly inactivates incretin hormones, limiting their plasma half lives to 2–5 min (Brubaker, 2007). Both these approaches have drawbacks, as mimetics need to be injected due to their peptidergic nature, while DPP-IV inhibition affects the processing of many other peptides, and for example interferes with the activation of peptide YY (PYY), another gut derived peptide that suppresses appetite in its active form (Ballantyne, 2006). Alternative approaches, such as the stimulation of intestinal L-cells, which secrete both GLP-1 and PYY, seem feasible, but would benefit from a greater understanding of the physiology of enteroendocrine cells.

Although GIP is believed to contribute at least as much as GLP-1 to the incretin effect under normal physiological conditions, the situation is complicated by the different insulinotropic efficiencies of GIP and GLP-1 in the context of diabetes. Thus, whilst pharmacological levels of GLP-1 or its DPP-IV resistant analogues undisputedly promote insulin secretion in type-2 diabetic patients, raising GIP levels is widely reported to be ineffective (Holst, 2007; Nauck et al., 1993). In a recent publication, GIP-receptor agonists were found still to increase insulin levels in subjects with newly diagnosed type-2 diabetes, but postprandial glucose excursions were not improved, possibly due to a simultaneous glucagonotropic effect (Chia et al., 2009). This is in marked contrast to the action of GLP-1-receptor agonists, which inhibit, rather than promote, glucagon secretion from the pancreatic α-cell (Vilsbøll, Krarup, Madsbad, & Holst, 2003). In addition there is increasing evidence that activation of the GIP-receptor has other undesirable effects, possibly acting in extrapancreatic locations such as adipocytes, where it promotes lipid storage (Kim, Nian, & McIntosh, 2007; Yip, Boylan, Kieffer, & Wolfe, 1998). Mice in which GIP-action is prevented by knocking out the GIP-receptor (Miyawaki et al., 2002), by treatment with a GIP-receptor antagonist (Gault et al., 2005, 2007) or by ablation of GIP-secreting cells (Althage et al., 2008), are somewhat protected from developing obesity on a high-fat diet or on the leptin-deficient, hence overeating, *ob/ob*-background. The leaner mice resulting from impaired GIP-signalling have better glucose tolerance than their fat littermates when assessed by an oral glucose tolerance test. As interference with GIP-action in non-obese mice worsens their glucose homeostasis (Gault et al., 2003; Miyawaki et al., 1999; Tseng, Kieffer, Jarboe, Usdin, & Wolfe, 1996), this might reflect the observation that GIPs incretin action seems to be impaired under chronic hyperglycaemic conditions, possibly due to fast downregulation of the GIP-receptor on pancreatic β-cells (Xu et al., 2007, Zhou et al., 2007). However, if the results from the rodent studies transfer to humans, it appears one would wish to stimulate GLP-1 secretion from L-cells, while simultaneously not affecting, or rather inhibiting, GIP secretion from K-cells. This might not easily be achievable as some cells in the proximal small intestine have been reported to contain both GLP-1 and GIP (Mortensen, Christensen, Holst, & Orskov, 2003), with ∼1/3 of all cells examined showing staining for both peptides (Theodorakis et al., 2006).

Until recently, very little was known about the molecular mechanisms underlying stimulus-secretion coupling in either K- or L-cells. Some conclusions about the nutrient sensing machinery could be drawn from studies in which incretin hormone release was measured in the whole body or upon luminal or vascular perfusion of isolated intestine (for review see Deacon, 2005). The drawback of these experiments is the uncertainty of whether the identified mechanisms are located within the incretin-secreting cells themselves or if they depend on communications with other cells, such as the surrounding enterocytes, neurons or paracrine influences from other enteroendocrine cells. Intracellular pathways in L- and K-cells have been postulated from the use of murine cell lines, such as the colonically derived GLUTag cell and the small intestinally derived STC-1 cell. Studies on primary cells have been limited by the inability to distinguish or isolate living enteroendocrine cells from the surrounding enterocytes. In this review I shall discuss recent insights into the sensing mechanisms for carbohydrate, lipids and protein characterised in incretin-secreting cell lines and more recently in primary K- and L-cells identifiable through the transgenic expression of the fluorescent protein Venus under the control of the GIP- and proglucagon-promoter, respectively.

GIP-secreting K-cells are found in the proximal small intestine, particularly the duodenum (Buchan, Polak, Capella, Solcia, & Pearse, 1979; Polak, Bloom, Kuzio, Brown, & Pearse, 1973), and are hence ideally placed to sense nutrient arrival in the gut. K-cell morphology, described as being “open-type” because of the microvillus-rich apical membrane directly facing the gut lumen, suggests that they might sense luminal contents directly. The mere presence of macronutrients in the gut lumen, however, is not sufficient to trigger GIP release, as pathophysiological (Besterman et al., 1978, 1979; Ebert & Creutzfeldt, 1980; Ebert, Creutzfeldt, Brown, Frerichs, & Arnold, 1976) or pharmacological (Fushiki, Kojima, Imoto, Inoue, & Sugimoto, 1992) inhibition of sugar absorption reduces GIP release in vivo.

The density of GLP-1 secreting L-cells, by contrast, increases along the length of the intestine, with the highest densities found in the colon and rectum, and only relatively low numbers located in the proximal small intestine (Eissele et al., 1992). It has hence been suggested that GLP-1 secretion in response to oral glucose does not involve direct sensing by L-cells, but rather reflects activation of upstream sensors which stimulate the distally located L-cells either by intestinal hormonal and/or neuronal loops (Rocca & Brubaker, 1999). However, L-cells are also open-type cells (Eissele et al., 1992), and L-cell model lines, like GLUTag, have been shown to respond to a number of nutrients, including glucose (Reimann & Gribble, 2002).

## 2 Carbohydrate sensing

As GIP-secreting cells were found to express glucokinase (hexokinase-IV), which has a restricted expression profile (Cheung et al., 2000; Jetton et al., 1994), it was hypothesised that glucose-sensing in K-cells must involve a mechanism similar to that employed by the pancreatic β-cell, where this enzyme plays a key role. Insulin secretion from β-cells is triggered in response to flux increases in glucose metabolism. *B*-cells express sufficient facilitative glucose transport capacity (GLUT-2) to allow rapid equilibration of the surrounding glucose concentration with that in the cytosol. Glucokinase, with its half maximal activity at ∼8 mm glucose, is well suited to monitor physiological changes in ambient glucose concentration, in contrast to the ubiquitously expressed hexokinases I–III, which become saturated at glucose concentrations in the range of ∼2–5 mm (Matschinsky, 2002). The resulting increased flux through glycolysis and the mitochondrial citric acid cycle results in a rise in the cytosolic ATP/ADP ratio. This in turn closes ATP-sensitive potassium channels (K_ATP_), leading to a net depolarization of the plasma membrane potential, which activates voltage gated Ca^2+^-channels. The consequent rise in cytosolic Ca^2+^ triggers vesicular fusion with the plasma membrane and release of stored insulin into the surroundings.

A single report investigated GIP secretion from canine epithelial cells in primary culture after partial enrichment for K-cells (to ∼10%) by elutriation (Kieffer, Buchan, Barker, Brown, & Pederson, 1994). In addition to glucose, elevation of the extracellular K^+^-ion concentration was found to be a potent secretory stimulus, thus establishing plasma membrane depolarization as a trigger for GIP release. However, direct glucose-sensing by K-cells has been disputed, as an STC-1 derived cell line did not increase its rate of secretion in response to either glibenclamide or glucose (Ramshur, Rull, & Wice, 2002), although other STC-1 derived cell lines have been shown to respond to glucose (Cheung et al., 2000; Kieffer et al., 1995). The lack of a sulphonylurea response reported by Ramshur et al. corresponded with other work by the same group failing to demonstrate colocalisation of K_ATP_-channel subunits and GIP in murine intestinal slices by immunohistochemistry (Wang, Chi, Li, Moley, & Wice, 2003). Nevertheless, expression of K_ATP_-channel subunits has since been demonstrated in GIP- and GLP-1-expressing cells in the human intestine (Nielsen et al., 2007), and the K_ATP_-channel opener diazoxide has been shown to decrease glucose-stimulated GIP release in dogs (Williams, 1979). Recently, expression of key glucose-sensing genes has been investigated in K-cells isolated by fluorescent assisted cell sorting (FACS) from transgenic mice expressing the fluorescent protein Venus under the control of the GIP-promoter (Parker, Habib, Rogers, Gribble, & Reimann, 2009). mRNAs for glucokinase and the K_ATP_-channel subunits Kir6.2 and SUR1 were found at levels comparable with those found in the pancreatic β-cell by quantitative PCR. Moreover, tolbutamide, a suphonylurea that closes K_ATP_-channels, triggered GIP release from mixed epithelial cultures isolated from mouse duodenum. However, while glucose responses in these cultures improved when cAMP-levels were pharmacologically raised, the cells became unresponsive to tolbutamide, suggesting that K_ATP_-channels are closed in K-cells at elevated cAMP concentrations and do not participate in glucose-sensing under these conditions.

**Fig. 1:**
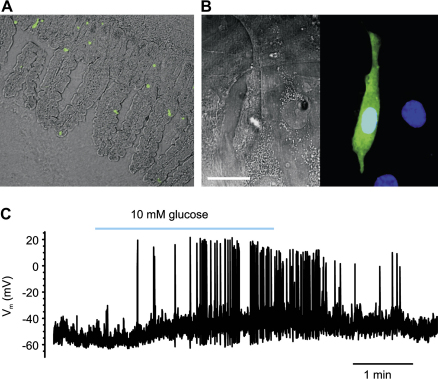
Glucose triggered electrical activity in L-cells. a) Venus-labeled L-cells in an ileal tissue slice. Phase contrast image of intestinal cells in an ileal slice taken from a mouse transgenic for the yellow fluorescent protein Venus under the control of the proglucagon-promoter. Venus-fluorescence was excited at 480 nm. b) Venus positive cells in a 7 day old colonic epithelial culture. *(left)* phase contrast, scale bar 20 μm *(right)* fluorescence image overlay, with Venus (green) and DAPI (blue). c) Current clamp recording from a Venus positive cell in colonic epithelial culture. The cell was in perforated whole-cell current clamp. Application of 10 mm glucose, as indicated by the bar, resulted in an increased action potential firing rate from ∼0.3 to ∼1.1 Hz (see Reimann et al., 2008 for methodological details).

**Fig. 2:**
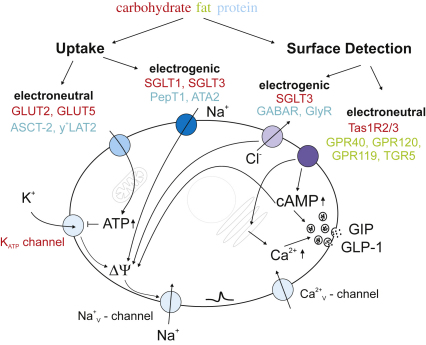
Nutrient sensing machinery in K- and L-cells. As in other endocrine cells, secretion is triggered by an elevation in cytosolic Ca^2+^, which promotes fusion of hormone containing vesicles with the plasma membrane. This can be achieved in different ways: (i) Uptake: (left) Electroneutral uptake of nutrients increases metabolic flux and elevates cytosolic ATP-levels. These close ATP-sensitive potassium channels (K_ATP_), permitting depolarization of the plasma membrane and opening of voltage gated sodium and calcium channels (Reimann et al., 2008), leading to an elevation in cytosolic Ca^2+^. (right) Electrogenic transport of nutrients directly depolarizes the plasma membrane and triggers all the subsequent steps. (ii) Alternatively nutrients might trigger hormone release without ever entering the cell by interaction with surface sensors. Examples for this type of detection involve activation of ligand gated ion channels (hence electrogenic (left) and feeding into the same triggering pathway as the depolarizing transport mechanisms) or (right) electroneutral activation of second messenger cascades. Examples of transporters/receptors identified in incretin-secreting cells are given for each of these mechanisms, with the colours corresponding to the macronutrients detected (see text for details).

Initial studies with canine L-cells in primary culture, isolated by elutriation, failed to detect glucose induced GLP-1 secretion (Damholt, Buchan, & Kofod, 1998). However, studies on the murine GLUTag cell line, which expresses GLP-1, but not GIP, have demonstrated responses to both glucose and tolbutamide (Reimann & Gribble, 2002). In contrast to the findings with K-cells in primary culture, tolbutamide stimulated GLP-1 release from GLUTag cells in the presence as well as the absence of raised cAMP-levels. RT-PCR detected the presence of glucokinase, Kir6.2 and SUR1 in GLUTag cells, and an increase in the ambient glucose concentration was shown to increase intracellular ATP-levels (Reimann, Williams, da Silva Xavier, Rutter, & Gribble, 2004). Studies with colonic L-cells, identified by the transgenic expression of the fluorescent protein Venus under the control of the proglucagon-promoter, confirmed many of the cell line findings (Reimann et al., 2008; Fig. 1). Primary L-cells were electrically active and increased their action potential frequency in response to either glucose or tolbutamide. This corresponded to an increase in cytosolic Ca^2+^, as monitored by Fura-2 fluorescence changes, and enhanced GLP-1 release in mixed epithelial cultures in response to either treatment, suggesting that glucose metabolism and subsequent K_ATP_-closure can trigger GLP-1 secretion (Fig. 2).

Despite the clear presence of K_ATP_-channels in human and mouse K and L cells, it seems that K_ATP_-channel closure does not, itself, act as the key link between elevated luminal glucose levels and incretin secretion. Mice lacking K_ATP_-channels, for example, were found to exhibit elevated, rather than suppressed, peak GIP levels after glucose ingestion, when compared with wild type controls (Miki et al., 2005). Furthermore, studies in humans have so far failed to detect significant changes in plasma GIP or GLP-1 in response to sulphonylureas (El-Ouaghlidi et al., 2007). To further address the role of the K_ATP_-channel in K- or L-cells it might be interesting to look at the incretin responses of patients with inactivating mutations in the K_ATP_-channel. These patients usually suffer from severe hypoglycaemia shortly after birth due to inappropriate hypersecretion of insulin from the pancreatic β-cell and consequently have often undergone lifesaving resection of the pancreas or are sometimes treated with diazoxide, a K_ATP_-channel opener. However, patients with K_ATP_-channel mutations, which increase the open probability of the channel at resting ATP-levels, have neonatal diabetes, due to defective insulin secretion from the pancreatic β-cell, but do not seem to have altered secretion of GLP-1 (Pearson et al., 2006). Similarly, patients with mutations in glucokinase, who suffer from maturity onset of diabetes in the young (MODY-2), appear to have normal incretin secretion (Murphy et al., 2009), suggesting that while the molecular machinery employed for glucose-sensing by the pancreatic β-cell is present, it does not seem to play as dominant a role in the stimulus-secretion coupling of either K- or L-cells.

An involvement of sodium-dependent glucose uptake in triggering incretin secretion has been suggested on the basis of the monosaccharide structure potency relationship in rodents and the sensitivity of glucose-stimulated GIP secretion to phloridizin (Ritzel, Fromme, Ottleben, Leonhardt, & Ramadori, 1997; Sykes, Morgan, English, & Marks, 1980). Sodium-coupled glucose transporter-1 (SGLT-1) has been shown to be expressed in the apical membrane of K-cells, and murine duodenal cultures secreted GIP in response to 10 mm α-methyl-glucopyranoside (α-MDG), a non-metabolisable glucose analogue transported by SGLT-1 (Parker et al., 2009). While the response to α-MDG was sensitive to phloridizin, as expected, it was also abolished by diazoxide, a K_ATP_-channel opener, suggesting that the mechanism involves depolarization of the plasma membrane, due to the coupled inflow of positively charged sodium ions. Consistent with this idea, α-MDG responses were only observed in the presence of raised cAMP-levels, when K_ATP_-channels appeared to be predominantly closed in these cells. In the GLUTag cell line, high concentrations of α-MDG (100 mm) increased action potential firing, and glucose-triggered electrical activity was inhibited in the additional presence of phloridizin (Gribble, Williams, Simpson, & Reimann, 2003). α-MDG (100 mm) triggered an inward current of ∼5 pA/cell, consistent with the typical magnitude of transporter currents, which was absent when extracellular Na^+^ was omitted. Smaller, but significant inward currents were recorded in response to 20 mm glucose. Expression of both SGLT-1 and SGLT-3a was detected by reverse transcription polymerase chain reaction (RT-PCR). It was subsequently reported that human, but not rabbit SGLT-3, lacks glucose transport capacity, and rather acts as a glucose gated ion channel (Diez-Sampedro et al., 2003). While this raises the interesting possibility that SGLT-3 acts a specific surface sensor for glucose, the fact that 3-O-methyl-glucose (3-OMG), which is a substrate for SGLT1 but not SGLT-3 (Díez-Sampedro, Lostao, Wright, & Hirayama, 2000; Voss, Díez-Sampedro, Hirayama, Loo, & Wright, 2007), triggers GLP-1 secretion (Reimann and Gribble, unpublished) suggests that the SGLT-3 transporter/sensor cannot, on its own, account for the observed incretin secretory responses to ingested sugars. Interestingly, primary epithelial cultures from murine small or large intestine secreted GLP-1 in response to 10 mm α-MDG even without pharmacological elevation of cytosolic cAMP-levels. As similar cultures secreted GLP-1 in response to tolbutamide, it seems that L-cells and K-cells may differ in their balance between the depolarizing SGLT-mechanism and counteracting resting K_ATP_ conductance. A recent publication investigated the effects of non-metabolisable glucose analogues on glucose homeostasis in mice in vivo (Moriya, Shirakura, Ito, Mashiko, & Seo, 2009). Not only was incretin secretion triggered by α-MDG and 3-OMG, but subchronic treatment of mice with 3% α-MDG in the drinking water for 13 days reduced the plasma glucose excursions in obese *db/db* mice in response to an oral glucose tolerance test. As this was observed without significant differences in body fat or weight, it suggests that chronic activation of sodium-coupled glucose transport might be a strategy to increase incretin secretion in the treatment of diabetes. An alternative explanation, however, could simply be a chronic reduction in the absorptive capacity of the small intestine and this needs to be further investigated. Further insight into the role of SGLT-1 in incretin secretion in humans might also be gained by studying patients harbouring mutations in the human SGLT-1 gene. These mutations, which either interfere with SGLT-1 transport capacity or its targeting to the brush border membrane, result in glucose/galactose malabsorption with severe diarrhoea, and are treated by exclusion of these sugars from the diet (Wright, Hirayama, & Loo, 2007). Incretin responses in patients lacking functional SGLT-1 have not, to my knowledge, been reported to date.

A third glucose-sensing mechanism identified in incretin-secreting cells involves G-protein coupled taste receptors. Taste receptor family 1 members 2 and 3 (Tas1R2 and Tas1R3) form relatively glucose sensitive heterodimers, which are found in sweet sensing papillae cells of the tongue (Chandrashekar, Hoon, Ryba, & Zuker, 2006). Expression of these receptors and the coupled G-protein gustducin have been reported in incretin-secreting cells (Dyer, Salmon, Zibrik, & Shirazi-Beechey, 2005; Rozengurt et al., 2006), and secretory responses to artificial sweeteners were demonstrated for example in the GLUTag and NCI-H716 cell lines, while gustducin knock-out animals showed impaired GLP-1 secretion (Jang et al., 2007; Margolskee et al., 2007). However, studies on primary murine L- and K-cells could barely detect Tas1R expression by quantitative PCR, and 1 mm sucralose, a saturating dose of this artificial sweetener on Tas1R2/3, did not significantly stimulate GIP or GLP-1 secretion from mixed intestinal epithelial cultures (Parker et al., 2009; Reimann et al., 2008). Subsequent *in vivo* studies could not detect significant increases in plasma incretin levels in response to artificial sweetener ingestion in rodents (Fujita et al., 2009; Moriya et al., 2009) or humans (Ma et al., 2009), although one study reported a significantly greater GLP-1 response to an oral glucose load, when diet-soda rather than carbonated water was consumed 30 min before the glucose stimulus (Brown, Walter, & Rother, 2009). However, as intestinal cells expressing Trpm5, a channel indispensable for taste perception through Tas1R signaling (Zhang et al., 2003), have now been reported not to express GLP-1 (Kokrashvili et al., 2009), the role of this pathway in glucose-triggered incretin secretion is far from clear.

## 3 Lipids

Due to their role in glucose homeostasis, much research has focused on the carbohydrate sensing of K- and L-cells. Whilst glucose ingestion results in a relatively rapid elevation of plasma GIP levels, fat ingestion triggers a greater and longer lasting GIP-response when compared on a molar basis (Falko, Crockett, Cataland, & Mazzaferri, 1975; Krarup, Holst, & Larsen, 1985). The molecular mechanisms underlying fat-stimulated GIP secretion, which under these somewhat artificial nutritional circumstances clearly does not function as an incretin, are only beginning to be understood and seem also be present in GLP-1 secreting L-cells. It has been reported that ingested triglycerides need to be hydrolysed to long-chain free fatty acids to stimulate GIP- or GLP-1 secretion (Ellrichmann et al., 2008; Enç et al., 2009; Kwasowski, Flatt, Bailey, & Marks, 1985; Pilichiewicz et al., 2003; Ross & Shaffer, 1981), and oils rich in monounsaturated fatty acid seem to be more potent than fats rich in saturated fatty acids (Lardonois, Starich, & Mazzaferri, 1988; Thomsen et al., 1999). One mechanism compatible with these observations has been described in the GLUTag cell line and involves activation of the atypical protein kinase C zeta (Iakoubov, Izzo, Yeung, Whiteside, & Brubaker, 2007). Long-chain unsaturated fatty acids are also ligands for the G_q_-protein coupled “free fatty acid receptors” GPR40 (Briscoe et al., 2003; Itoh et al., 2003) and GPR120 (Hirasawa et al., 2005). mRNAs for both these receptors are detected by quantitative PCR in L- and K-cells (Parker et al., 2009; Reimann et al., 2008), as well as in pancreatic α- and β-cells (unpublished). Knock-down of GPR120, but not GPR40, interfered with CCK-release from STC-1 cells (Tanaka et al., 2008) and selective GPR120 agonists, but not selective GPR40-agonists, triggered secretion of GLP-1 from this cell line (Hara et al., 2009). Short chain fatty acids, products of bacterial fermentation in the colon, have been shown to stimulate the related G-protein coupled receptors GPR41 and GPR43, which have been localized immunohistochemically on L-cells in rat and human (Karaki et al., 2006; Tazoe et al., 2008). Other compounds elevated in the gut lumen after a lipid rich meal, such as lysophosphatidylcholine, oleoylethanolamide or bile acids, have been shown to stimulate the G_s_-protein coupled receptors GPR119 and TGR5, respectively. GPR119 agonists stimulate GIP and GLP-1 secretion in vivo (Chu et al., 2008; Lauffer et al., 2009), while TGR5 activation has been shown to stimulate GLP-1 secretion from STC-1 cells. Both GPR119 and TGR5 knock-out animals showed attenuated postprandial GLP-1 secretion, but did not exhibit significantly altered glucose homeostasis, even when challenged with a high-fat diet (Chu et al., 2008; Lan et al. 2009; Thomas et al., 2009). Similarly, GPR40 knock-out animals showed significantly reduced plasma GIP and GLP-1 levels on a high-fat diet, yet GPR40^−/−^ mice are not significantly protected from developing diet induced obesity, and different laboratories have reported inconsistent effects on glucose homeostasis in high-fat fed mice (Edfalk, Steneberg, & Edlund, 2008; Kebede et al., 2008; Lan et al., 2008; Steneberg, Rubins, Bartoov-Shifman, Walker, & Edlund, 2005). Obviously, effects on glucose homeostasis are complicated because of the expression of all the above-mentioned GPRs on both gut enteroendocrine and pancreatic islet cells and cross talk between these cells, as for example insulin has itself been reported to stimulate GLP-1 secretion (Lim et al., 2009). Further problems arise from possible “lipotoxic” effects due to chronic activation of these receptors, which might counteract their acute stimulatory effects on hormone secretion (Alquier & Poitout, 2009; Steneberg et al., 2005). The recent availability of more selective pharmacological tools, possibly in combination with conditional knock-out technology, should clarify the importance of these GPRs for lipid stimulated incretin secretion, and enable an assessment of the relative contribution of the incretin effect to the global metabolic outcome of targeting these receptors therapeutically.

## 4 Protein

Protein is usually considered the least effective of the macronutrients at stimulating incretin secretion. However, intraluminal administration of amino acids (Thomas et al., 1976, 1978) or acidification (LeRoith et al., 1980) have been shown to stimulate GIP release. Furthermore, meat hydrolysate is reported to be one of the strongest stimuli of GLP-1 secretion from the human NCI-H716 cell line (Reimer et al., 2001) and also stimulates GIP and GLP-1 secretion from STC-1 and GLUTag cells (Cordier-Bussat et al., 1998). The mechanism for this is unclear, but secretion can be inhibited by drugs targeting voltage gated Ca^2+^-channels and hence presumably involves plasma membrane depolarization (Ramshur et al., 2002), which could be achieved by electrogenic co-transport of small peptides and protons by the PepT1 transporter. However, in the STC-1 cell line this could only be demonstrated after expression levels of PepT1 were boosted by transfection (Matsumura, Miki, Jhomori, Gonoi, & Seino, 2005) and alternatively an activation of mitogene-activated protein kinases has been suggested (Reimer, 2006). The amino acid glutamine was recently found to stimulate GLP-1 secretion in control, over weight and diabetic human volunteers (Greenfield et al., 2009). Glutamine also stimulates GIP secretion from mouse duodenal cells in primary culture (Parker et al., 2009). The underlying mechanism has been investigated in the GLUTag cell line and was at least in part dependent on the electrogenic uptake of the amino acid, presumably via a system A transporter (Reimann et al., 2004). Alanine and glycine by contrast stimulated GLP-1 secretion from the GLUTag cell line due to the activation of the ionotropic GABA-receptor, a chloride channel, opening of which resulted in strong depolarization (Gameiro et al., 2005).

## 5 Summary and conclusion

GIP-secreting K-cells and GLP-1 secreting L-cells seem to employ similar molecular sensors to detect the arrival of macronutrients in the gut. Coupling of nutrient transport with the electrogenic uptake of ions provides a direct mechanism to depolarize the cell plasma membrane, thus enabling non-metabolisable nutrient analogues to stimulate secretion from these cells. Lipids seem to act at least partly via activation of specific G-protein coupled receptors, which trigger signaling cascades rising cytosolic Ca^2+^ and cAMP. Identification of the specific transporters and receptors involved might allow the manipulation of endogenous incretin pools in the treatment of obesity and diabetes.
